# The subfamily Cheloninae (Hymenoptera, Braconidae) from Egypt, with the description of two new species

**DOI:** 10.3897/zookeys.115.1186

**Published:** 2011-07-05

**Authors:** Yusuf Abd-Elaziz Edmardash, Mahmoud Saleh Abdel-Dayem, Neveen Samy Gadallah

**Affiliations:** Entomology Department, Faculty of Science, Cairo University, Giza, Egypt

**Keywords:** Cheloninae, Braconidae, Hymenoptera, Egypt, new species

## Abstract

A key to the chelonine species (Braconidae) (both recorded and recently collected) from Egypt is given. It includes 16 species, of which five species are new to the Egyptian fauna and two (*Phanerotoma (Phanerotoma) elbaiensis* **sp. n.** and *Phanerotoma (Bracotritoma) ponti* **sp. n.**) are new for science. A faunistic list and the description for the two new species are added.

## Introduction

Cheloninae is a moderately large subfamily within the important parasitoid family Braconidae. The subfamily comprises more than 1300 described species worldwide (Yu et al. 2005). Members of this subfamily are present in all zoogeographical regions. Inspite of their worldwide distribution, only the tribes Chelonini and Phanerotomini are represented in the Palaearctic fauna (van Achterberg 1990, Yu et al. 2005, Aydogdu 2008).

Chelonines are small to medium-sized wasps (1.8–6.0 mm long), with a rigid non-articulated metasomal carapace which is formed by the fusion of the first three metasomal tergites. This carapace is open ventrally and encloses the soft parts of the metasoma.

Wasps of the subfamily Cheloninae are known to be solitary egg-larval endoparasitoids of many lepidopterous families, and may be considered as potential bio-control agents (Walker and Huddleston 1987, Inayatullah and Naeem 2004).

In Egypt, very little attention has been paid to the taxonomy of this group of parasitoids despite their potential importance as biocontrol agents. The first work mentioning some chelonines from Egypt was that of Szépligeti (1908) who recorded two species (*Ascogaster excisa* (Herrich-Schäffer, 1838) and *Chelonus basalis* Curtis, 1837). Five other species (*Chelonus blackburni* Cameron, 1886, *Chelonus sulcatus* Jurine, 1807, *Phanerotoma dentata* (Panzer, 1805), *Phanerotoma hendecasisella* Cameron, 1905 and *Phanerotoma leucobasis* Kriechbaumer, 1894 [as *Phanerotoma ocularis* Kohl, 1906]) were listed for the Egyptian fauna by Shenefelt (1973). In 1976, Morsy recorded five species including those of Szépligeti but only *Chelonus sulcatus* Jurine, 1807 and *Phanerotoma dentata* (Panzer, 1805)from Shenefelt’s catalogue, in addition to *Chelonus inanitus* (Linnaeus, 1767). Finally, two chelonine species (*Phanerotoma leucobasis* Kriechbaumer, 1894 and *Phanerotoma masiana* Fahringer, 1934) were listed by van Achterberg (1990) for Egypt, thus raising the total number to nine species.

In the present paper, a key is presented for 16 chelonine species collected and recorded from different regions of Egypt in the period between 2008 and 2010. Five species are recorded for the first time in Egypt, and two are new species: *Phanerotoma (Phanerotoma) elbaiensis* sp. n.and *Phanerotoma (Bracotritoma) ponti* sp. n.

## Material and methods

Regular surveys of chelonine wasps were undertaken from the beginning of 2008 to the end of 2010, covering various regions of Egypt. Sampling was done by means of net sweeping and light trapping.

Morphological terms and wing venation terminologies are based on van Achterberg (1988, 1993); body sculpture terminology is based on Harris (1979). Drawings were made using a camera lucida attached to an Olympus stereo-microscope (SZX9). Measurements were made using an ocular micrometer.

Global distribution and synonyms of the listed species are based mainly Yu et al. (2005).

The characters of the tribes, genera and subgenera (of genus *Phanerotoma*) in the key are based on van Achterberg (1990). New records are marked with an asterisk.

**Abbreviations:** M= medial vein; OOL= ocellocular line; POL= posterior ocellar line; R= radial vein; r= transverse radial vein; SR= radial sector vein; T= metasomal tergite.

**List of depositories:**

ASUCAin Shams University collection

CUCCairo University collection

MACMinistry of Agriculture collection

ESECEntomological Society of Egypt collection

### Key to the chelonine species from Egypt

**Table:** 

1	Metasoma without distinct transverse sutures (Figs 19, 22); body usually dark brown or black (Tribe Chelonini Foerster, 1862)	2
–	Metasoma with two distinct sutures (Figs 4, 36, 44); body usually yellowish-brown (Tribe Phanerotomini Baker, 1926; genus *Phanerotoma* Wesmael, 1838)	10
2	Vein 1-SR+M of fore wing present (Fig. 9); male carapace without apical aperture; vein r of fore wing usually arises far distad of middle of pterostigma (Fig. 23) (Genus *Ascogaster* Wesmael, 1835)	3
–	Vein 1-SR+M of fore wing absent (Fig. 10); male carapace with or without apical aperture; vein r of fore wing arises near middle of pterostigma (Figs 10, 24) (Genus *Chelonus* Panzer, 1806)	4
3	Propodeum with four sharp medium-sized teeth posteriorly (Fig. 7); hind tibia entirely black except basally; carapace 0.8 times length of head and mesosoma combined	*Ascogaster quadridentata* Wesmael, 1835
–	Propodeum with two small teeth posteriorly (Fig. 8); hind tibia entirely brown except apically; carapace as long as head and mesosoma combined	*Ascogaster excisa* (Herrich-Schäffer, 1838)
4	Female antenna always with 16 flagellomeres, male with more than 16 flagellomeres; male carapace with apical aperture (Figs 12, 13); length of body not exceeding 3.6 mm. (Subgenus *Microchelonus* Szépligeti, 1908)	5
–	Antenna of both sexes with more than 16 flagellomeres; male carapace without apical aperture; carapace usually with two subbasal yellowish spots; length of body exceeding 4.4 mm. (Subgenus *Chelonus* Panzer, 1806)	8
5	Female carapace with yellowish basal band extending to half its length (Fig. 11), entirely black in male; male apical aperture small and rounded, not exceeding half width of carapace (Fig. 12); body length 1.8 or 1.9 mm	*Chelonus basalis* Curtis, 1837
–	Carapace of both sexes entirely black or with yellowish, whitish or ivory subbasal band usually extended to about half (slightly longer or shorter) of its length; male apical aperture slit-like, transversely elongated, significantly exceeding half width of carapace (except for *Chelonus blackburni*) (Fig. 13); body length 2.8–3.6 mm	6
6	Carapace entirely black, coarsely longitudinally rugose; male antenna with 23 flagellomeres	*Chelonus sulcatus* Jurine, 1807
–	Carapace with whitish (or ivory) subbasal band of variable length, densely reticulate; male antenna with 25–26 flagellomeres (only for *Chelonus curvimaculatus*)	7
7	Maximum length of female carapace about 2.1 times its maximum height; POL as long as (or very slightly longer than) OOL (Fig. 16); coloured band of carapace mostly extended to 0.4 of its length (Fig. 14)	*Chelonus blackburni* Cameron, 1886
–	Maximum length of female carapace 2.4–2.7 times its maximum height; POL 0.6–0.7 times OOL (Fig. 17); coloured band of carapace usually extended to half (or slightly more) of its length (Fig. 15)	*Chelonus curvimaculatus* Cameron, 1906
8	Vertex with weak transverse striae behind ocelli; maximum length of female carapace 2.3 times its maximum height (Fig. 19); POL 1.5–1.6 times as long as OOL (Fig. 18)	*Chelonus obscuratus* Herrich-Schäffer, 1838
–	Vertex with coarse transverse striae behind ocelli (Fig. 20); maximum length of female carapace 2.6–2.9 its maximum height (Fig. 22); POL 1.1–1.2 times as long as OOL (Fig. 20)	9
9	Ovipositor thick (Fig. 21); vein r of fore wing distinctly angled with vein 3-SR (Fig. 24); vein 1-M of fore wing yellowish; yellowish spots of carapace usually more or less rounded and may be absent; body length 5.2–6.0 mm	*Chelonus inanitus* (Linnaeus, 1767)
–	Ovipositor slender (Fig. 22); vein r of fore wing nearly linear with 3-SR (Fig. 25); vein 1-M of fore wing dark brown; yellowish spots of carapace usually more or less quadrate; body length 4.4–5.1 mm	*Chelonus oculator* (Fabricius, 1775)
10	Maximum width of pterostigma 1.1–5.3 times length of vein 3-SR (in the Egyptian specimens 1.7–4.1 times); vein r of fore wing 1.0–2.1 times length of vein 3-SR (Figs 1, 31) (Subgenus *Bracotritoma* Csiki, 1909)	11
–	Maximum width of pterostigma 0.5–1.1 times length of vein 3-SR ((in the Egyptian specimens 0.5–0.8 times); vein r of fore wing 0.1–0.5 times length of vein 3-SR (Figs 3, 9, 27) (Subgenus *Phanerotoma* Wesmael, 1838)	12
11	Vein r of fore wing about 2.1 times as long as 3-SR (Fig. 1); scape 1.5 times as wide as first antennal flagellomere (Fig. 2); vein 1-M and parastigma pale yellow; length of eye in dorsal view 2.7 times temple	*Phanerotoma masiana* Fahringer, 1934
–	Vein r of fore wing as long as 3-SR (Fig. 31); scape slightly wider than first antennal flagellomere (Fig. 32); vein 1-M and parastigma dark brown; length of eye in dorsal view nearly twice as long as temple (Fig. 29)	*Phanerotoma ponti* sp. n.
12	Metasomal T3 truncate posteriorly, with protruding corners posteriorly (Figs 28, 44)	13
–	Metasomal T3 rounded posteriorly, without protruding corners posteriorly (Figs 4, 36)	14
13	Parastigma yellowish except basally; length of eye in dorsal view 1.9 times as long as temple (Fig. 38); vein r of fore wing 0.5 times length of vein 3-SR (Fig. 39)	*Phanerotoma elbaiensis* sp. n.
–	Parastigma dark brown; length of eye in dorsal view 1.1 times as long as temple (Fig. 26); vein r of fore wing 0.2 times length of vein 3-SR (Fig. 27)	*Phanerotoma rufescens* (Latreille, 1809)
14	Veins SR1 and 2-SR straight or nearly so (Fig. 3)	*Phanerotoma dentata* (Panzer, 1805)
–	Vein SR1 nearly straight and vein 2-SR obviously curved (Figs 6, 9)	15
15	Vein 2-SR slightly bent; vein r of fore wing 0.2–0.3 times as long as vein 3-SR (Fig. 9)	*Phanerotoma leucobasis* Kriechbaumer, 1894
–	Vein 2-SR distinctly bent; vein r of fore wing 0.1 times as long as vein 3-SR (Fig. 6)	*Phanerotoma hendecasisella* Cameron, 1905

## Checklist of the Egyptian chelonine species

### Tribe Chelonini Nees

#### Genus Ascogaster Wesmael, 1835

*Ascogaster* Wesmael, 1835: 226. Type-species: *Ascogaster instabilis* Wesmael. Designated by Foerster 1862.

##### Ascogaster excisa

(Herrich-Schäffer, 1838)

http://species-id.net/wiki/Ascogaster_excisa

Chelonus excisus Herrich-Schäffer 1838: 153, ♀.
Ascogaster longiventris Tobias, 1964: 148, ♂.

###### Distribution.

Egypt [without specific locality (Szépligeti 1908); Alexandria (Morsy 1976), Bulgaria, France, Germany, Kazakhstan, Russia, Spain, Switzerland and former Yugoslavia.

##### * Ascogaster quadridentata

Wesmael, 1835

http://species-id.net/wiki/Ascogaster_quadridentata

Ascogaster quadridentata Wesmael 1835: 237.

###### Material.

1♀, Arish (31°8'11.148"N; 33°49'57.5754"E), 14.III.2009 [CUC].

###### Distribution.

New to Egypt,Europe (Central, Southeast and Western), Mongolia, New Zealand [introduced (Walker and Huddleston 1987)], North Africa and Russia (Central, Northwest and South).

#### Genus *Chelonus* Panzer, 1806

*Chelonus* Panzer, 1806: 164. Type-species: *Ichneumon oculator* Fabricius. (Monobasic).

##### Chelonus (Chelonus) inanitus

(Linnaeus, 1767)

http://species-id.net/wiki/Chelonus_(Chelonus)_inanitus

Cynips inanita Linnaeus 1767: 919.>

###### Material.

1♀, Alexandria (31°12'58.248"N; 29°45'58.248"E), VI.1965 [ESEC]; 1♀, 2 ♂♂, Assuit (27°16'3.756"N; 31°9'6.9834"E - 27°23'29.1834"N; 31°32'26.484"E), IX.1972 [ASC]; 1♀, Beni-Suef (29°13'59.9874"N; 31°1'0.012"E), IX.1972 [ASC]; 2 ♀♀, Kerdasa (30°1'56.136"N; 31°6'32.6874"E), 29.X.2008 [CUC]; 2 ♀♀, 1 ♂, Nahia (30°1'55.2354"N; 31°6'39.4194"E), 28.X.2008 [**CUC**]; 1♀, 2 ♂♂, Ismailia (30°32'54.168"N; 31°47'0.2754"E - 30°38'20.7954"N; 32°16'7.572"E), 25.XI.2009 [CUC]; 1♀, 1♂, Fayoum [Karanis] (29°21'N; 30°40'59.988"E), 23.VIII.2010 [CUC].

###### Distribution.

Egypt [El-Menia, El-Sharqia (El-Zagazig and Menia El Qamh), Gharbia, Sheiben El Kom and Qena (Morsy 1976)], Europe, Israel, Japan, North Africa, Russia and USA [California, introduced (Shenefelt 1973)].

##### * Chelonus (Chelonus) obscuratus

Herrich-Schäffer, 1838

http://species-id.net/wiki/Chelonus_(Chelonus)_obscuratus

Chelonus obscuratus Herrich-Schäffer 1838: 154.

###### Material.

1♀, 1 ♂, El- Menia (28°29'18.96"N; 30°50'55.8954"E), 19.VII.1974 [**ASC**]; 2♀♀, Borg el Arab (30°52'9.4434"N; 29°24'44.8194"E), 13.IV.2009 [CUC]; 1♀, Matruh (31°36'54.3234"N; 25°55'35.2554"E), 30.IX.2009 [CUC].

###### Distribution.

**N**ew to Egypt, Europe, Mongolia, North Africa and Russia (Central, East and South)

##### * Chelonus (Chelonus) oculator

(Fabricius, 1775)

http://species-id.net/wiki/Chelonus_(Chelonus)_oculator

Ichneumon oculator Fabricius 1775: 338.

###### Material.

1♂, 1♀ Damanhour (31°1'59.9874"N; 30°28'0.012"E) 4.XI.2008 [CUC]; 1♀ Banha (30°27'27.4314"N; 31°10'12.42"E) 15.X.2009 [CUC]; 1♀ Desouq (31°7'47.1"N; 30°38'45.3834"E) 29.XII.2009 [CUC].

###### Distribution.

New to Egypt,Europe, Mongolia and Russia.

##### Chelonus (Microchelonus) basalis

Curtis, 1837

http://species-id.net/wiki/Chelonus_(Microchelonus)_basalis

Chelonus (Microchelonus) basalis Curtis 1837: 672.

###### Distribution.

In Egypt previously recorded with no specific locality (Szépligeti 1908),Europe (Central and Southwest), Israel, Russia (Northwest) and West Asia.

##### Chelonus (Microchelonus) blackburni

Cameron, 1886

http://species-id.net/wiki/Chelonus_(Microchelonus)_blackburni

Chelonus carinatus Cameron 1881: 599 (not Provancher, 1881).
Chelonus blackburni Cameron 1886: 242, replacement name.
Chelonus cameronii Dalla Torre 1898: 200, replacement name for *carinatus* Cameron.>

###### Material.

2♀♀, Ismailia (30°24'16.2354"N; 32°17'38.868"E ), 20.IV.2008 [CUC]; 1♀, El Tal el-kabeir (30°32'54.168"N; 31°47'0.2754"E ), 21.IV.2008 [CUC]; 1♀, Ras El-esh (31°45'15.2"N; 32°18'30.008"E ), 17.V.2010 [CUC].

###### Remarks.

The extreme basal part of the carapace has a black bilobed (kidney-shaped) marking, but in some specimens it may be semi-circular; the hind tibia has a whitish median band which in some cases is weakly developed.

###### Distribution.

Egypt [introduced (Shenefelt 1973)]**,** Australia, Fiji [introduced (Shenefelt 1973)], Hawaii, Kure Island, Mexico, Puerto Rico [introduced (Shenefelt 1973)] and USA [Texas, introduced and not established (Shenefelt 1973).

##### * Chelonus (Microchelonus) curvimaculatus

Cameron, 1906

http://species-id.net/wiki/Chelonus_(Microchelonus)_curvimaculatus

Chelonus curvimaculatus Cameron 1906: 34.

###### Material.

2♀♀, 1 ♂, Sonnores (29°24'55.0434"N; 30°51'54.108"E ), 11.XI.2008 [**CUC**]; 1♀ Ebshwai (29°21'58.6074"N; 30°40'57.8274"E ), 11.XI.2008 [CUC]; 1♀, Tahta (26°46'1.6314"N; 31°29'44.1954"E ), 20.X.2009 [CUC]; 1♀, 1♂, Armant (25°37'20.3154"N; 32°32'33.936"E ), 17.XII.2009 [CUC].

###### Remarks.

The subbasal ivory band of the carapace is usually curved or rounded apically; in one specimen it is more or less V-shaped.

###### Distribution.

New to Egypt,Africa (North- and Southeast), Congo and Senegal.

##### Chelonus (Microchelonus) sulcatus

Jurine, 1807

http://species-id.net/wiki/Chelonus_(Microchelonus)_sulcatus

Chelonus sulcatus Jurine 1807: 291.

###### Material.

1 ♀, Menia El Kamh (30°30'55.404"N; 31°20'58.02"E ), 12.XI.1973 [ASC]; 3 ♀♀, 1 ♂, Arab El Raml (31°14'35.0514"N; 29°57'36.756"E ), 4.III.1975 [ASC]; 1♀, Samanoud (30°57'35.928"N; 31°14'15.8994"E ), X.1981 [MAC]; 1 ♀, Beba (28°55'25.4274"N, 30°59'2.2914"E ), 25.IV.2008 [CUC].

###### Remarks.

The examined specimens have the carapace entirely black, but in a single specimen (from Samanoud), a peculiar crown-shaped basal orange reddish spot is present.

###### Distribution.

Egypt [Beni Suef, Minya and Sids (Morsy 1976)],Europe, Israel, Mongolia and Russia.

### Tribe Phanerotomini Baker

#### Genus *Phanerotoma* Wesmael, 1838

*Phanerotoma* Wesmael, 1838: 165. Type-species: *Chelonus dentatus* Panzer. Designated by Haliday, 1804 in Westwood.

##### Phanerotoma (Bracotritoma) masiana

Fahringer, 1934

http://species-id.net/wiki/Phanerotoma_(Bracotritoma)_masiana

Phanerotoma (Bracotritoma) masiana Fahringer 1934: 573.

###### Material.

1 ♀, Arish (31°8'5.028"N; 33°48'40.752"E ), 15.VII.1980 [CUC].

###### Distribution.

Egypt [Sinai-Wadi Isla, Khammissa (van Achterberg 1990)]**,** Libya and Saudi Arabia.

##### Phanerotoma (Bracotritoma) ponti sp. n.

urn:lsid:zoobank.org:act:F999AA12-8376-4F61-A323-9033A4DD0031

http://species-id.net/wiki/Phanerotoma_(Bracotritoma)_ponti

###### Description.

**(Figs 29–37)** ♀: Length of body: 3.4 mm. Length of fore wing: 2.4 mm.

Colour: Generally yellowish-brown, with the following parts dark brown to black: stemmaticum, mesoscutum (especially laterally), metanotum, sides of scutellum, propodeum posteriorly, third metasomal tergite (T3) (except laterally), pterostigma (except basal and apical 0.2), parastigma, apex and sub-basal part of middle tibia (except extreme apex which is paler), apical 0.3 and subbasal ring of hind tibia, apical half of hind basitarsus and telotarsus; apical eight antennal flagellomeres, tegula and humeral plate slightly pale brown; vein 1-M slightly paler than parastigma; apical third of fore wing infuscate; middle tibia whitish medially and basally.

Head: Slightly wider than maximum width of mesosoma; eyes slightly divergent below; preapical antennal flagellomeres cylindrical, slightly narrowed basally, apical flagellomere 1.1 times length of preapical one, scape slightly wider than first flagellomere; vertex and frons with fine transverse striae; face nearly smooth; inner tooth of mandible slightly shorter than outer tooth; length of eye in dorsal view nearly twice as long as temple; POL twice diameter of posterior ocellus; POL 0.6 times OOL; length of malar space 0.7 times basal width of mandible; longitudinal eye diameter as long as transverse diameter.

Mesosoma: Mesoscutum finely granulated; propodeum finely punctate. Fore wing with vein r as long as vein 3-SR; maximum width of pterostigma 1.7 times vein 3-SR; veins 2-SR and 1-SR straight; middle tibia without distinct blister; outer hind tibial spur 1.1 times the inner one and 0.3 times basitarsus, basitarsus about 0.9 times as long as following tarsomeres combined.

Metasoma: Ovoid, more or less parallel-sided, narrowed posteriorly; metasomal T1 and T2 with irregular fine longitudinal striae, T3 with dense reticulations, its maximum length slightly more than 1.3 times of that of T2; ovipositor not protruding beyond apex of metasoma.

**Male:** Unknown.

###### Diagnosis.

This species is closely related to *Phanerotoma (Bracotritoma) bouceki* van Achterberg, but *ponti* hasthe parastigma dark brown, vein 1-M slightly paler than in *bouceki*, middle tibia is darker and the blister of the middle tibia is much less apparent than in *bouceki*.

###### Etymology.

This species is named in the honour of Dr. Adrian Pont (Oxford University Museum of Natural History, UK).

###### Type Material.

Holotype, ♀, Gabal Elba – El Shallal (22°2'59.604"N; 36°32'4.2"E ), 15.II.2010. [CUC].

##### Phanerotoma (Phanerotoma) dentata

(Panzer, 1805)

Chelonus dentatus Panzer 1805: 88.

###### Material.

2♀♀, 1♂ Abu Rawash (30°3'13.86"N; 31°4'36.0834"E ), 11: 12.IX.1932 [**MAC**]; 2♀♀, Banha (30°27'27.4314"N; 31°10'11.676"E ), 18.VIII.1972 [**ASC**]; 1♀, 2♂♂, Cairo (29°57'18.684"N; 29°57'18.684"E ), 6.V.1975 [**ASC**]; 2♀♀, Wadi El Natroun (30°29'57.6024"N; 29°58'54.177"E ), 14 X .2009 [**CUC**].

###### Distribution.

Egypt [without specific locality, (Shenefelt 1973); Alexandria (Abd-Rabou 2008)]**,** Europe, Israel, Japan, Kenya, Korea and Russia (East, Northwest and South) USA [California, introduced (Shenefelt 1973)].

##### Phanerotoma (Phanerotoma) elbaiensis sp. n.

urn:lsid:zoobank.org:act:830B2EDF-9596-473C-8CCB-BB9474C3728C

http://species-id.net/wiki/Phanerotoma_(Phanerotoma)_elbaiensis

###### Description.

**(Figs 38–45)** ♀: Length of body: 4.1mm. Length of fore wing: 3.5mm.

Colour: Generally yellowish-brown with black stemmaticum; the following parts are dark-brown: shiny scape, seven apical antennal flagellomeres, lateral margin of mesoscutum, sides of scutellum, medio-posterior depression of scutellum, lateral sides of first metasomal tergite (T1), lateral side and a central rounded spot on T2, entire T3, apical half of middle tibia, apical 0.3 as well as subbasal ring of hind tibia, apical half of hind basitarsus and about the basal 0.7 of the other tarsomeres; tegula, humeral plate, pterostigma (except basal 0.3) and vein 1-M. Veins 1-R1 and 2-SR pale yellow; parastigma yellowish (but brown basally).

Head: Slightly wider than maximum width of mesosoma; eyes slightly divergent above and below; preapical antennal flagellomeres moderately moniliform, apical flagellomere 1.3 times length of preapical one, scape 3 times as wide as first flagellomere; vertex smooth and shiny medially, weakly rugose laterally; frons rugose but much coarser than vertex; face densely and finely punctate laterally, with fine transverse rugulae medially; inner tooth of mandible slightly less than half as long as outer tooth; length of eye in dorsal view about 1.9 times temple; POL 0.5 times diameter of posterior ocellus; POL 0.3 times OOL; length of malar space 0.8 times basal width of mandible; longitudinal eye diameter slightly longer than transverse diameter.

Mesosoma: Finely and densely punctate; mesoscutum coarsely striated laterally (near base of fore wing); metanotum smooth and shiny; propodeum with very fine longitudinal rugae that are curved towards its center and become transverse and much coarser postero-medially. Vein r of fore wing 0.5 times 3-SR; maximum width of pterostigma 0.6 times 3-SR; 2-SR and 1-SR are nearly straight; middle tibia with distinct blister; outer hind tibial spur 1.2 times as long as inner one, slightly longer than 0.3 times basitarsus; hind basitarsus about 0.6 times the following tarsomeres combined.

Metasoma: Ovoid, truncate posteriorly; T1 and T2 with irregular longitudinal reticulation, much denser and curved on complete T3; T3 with protruding corners latero-posteriorly and excavated posteriorly; maximum length of T3 slightly more than 1.1 times maximum length of T2; ovipositor greatly protruding beyond apex of metasoma; hypopygium modified, with a relatively small apical spine.

**Male:** Unknown.

###### Diagnosis.

This species is related to *Phanerotoma (Bracotritoma) bilinea* Lyle, but *elbaiensis* hasthe middle tibia with a distinct blister; the parastigma brownish basally; the vein 1-M darker and the apical spine of the hypopygium relatively small. It is similar to *Phanerotoma (Bracotritoma) maculata* (Wollaston), especially because of the long protruding ovipositor, but differs by its general colour, especially by the yellowish parastigma and basal third of pterostigma (dark brown in *maculata*). In addition, the characters of the subgenus *Phanerotoma*, to which the new species belongs, are different.

###### Etymology.

The species name *elbaiensis* refers to its type locality (Gabal Elba).

###### Type material.

Holotype, ♀, Gabal Elba – Wadi Aeibed (22°19'28.092"N; 36°25'24.636"E ), 27.I.1982. [CUC].

##### Phanerotoma (Phanerotoma) hendecasisella

Cameron, 1905

http://species-id.net/wiki/Phanerotoma_(Phanerotoma)_hendecasisella

Phanerotoma hendecasisella Cameron 1905: 80.

###### Material.

2♀♀, Alexandria (31°11'0.42"N; 29°56'44.304"E ), 14.V.1980 [**MAC**]**;** 1♀, Tanta (30°54'2.6634"N; 31°9'50.8386"E ), without date [**CUC**].

###### Distribution.

Egypt (with no specific locality, Shenefelt 1973),Australia, Burma, Ceylon and India.

##### Phanerotoma (Phanerotoma) leucobasis

Kriechbaumer, 1894

http://species-id.net/wiki/Phanerotoma_(Phanerotoma)_leucobasis

Phanerotoma leucobasis Kriechbaumer 1894: 62.

###### Material.

5♀♀, 1♂ Kerdasa (30°1'56.136"N; 31°6'32.6874"E ), II.1965 [**MAC**]; 3♀♀, El Mansouria (30°8'14.1"N; 31°3'46.656"E ), IX.1995 [**MAC**]; 4♀♀, 1 ♂ Safaga (26°44'25.764"N; 33°58'54.5514"E ), VII.2007 [**CUC**]; 2♀♀, 1 ♂ Giza (29°37'18.048"N; 31°15'14.508"E ), 13.II.2008 [**CUC**]; 1♀, Ismailia (30°38'20.7954"N; 32°16'7.572"E ), 28.III.2008 [**CUC**]; 1♀, 1♂ Alexandria (31°5'1.3914"N; 29°45'53.316"E ), 4.X.2008 [**CUC**]; 2♀♀, 1♂ Assuit (31°32'55.248"N; 27°23'33.2514"E ), 12.I.2009 [**CUC**].

###### Variation.

The colour of the head varies from yellowish brown to nearly black; the terminal flagellomeres are usually blackish, but in few cases paler; the third metasomal tergite is usually brownish, but blackish in a few specimens, and the second tergite sometimes has brownish spots laterally.

###### Distribution.

In Egypt previously recorded from Alexandria, Dokki, Gara, Maadi, Sinai (van Achterberg 1990), Africa (Central, North and Southeast), Israel, USA [California, introduced (Shenefelt 1973)] and West Asia

**Figures 1–13.**
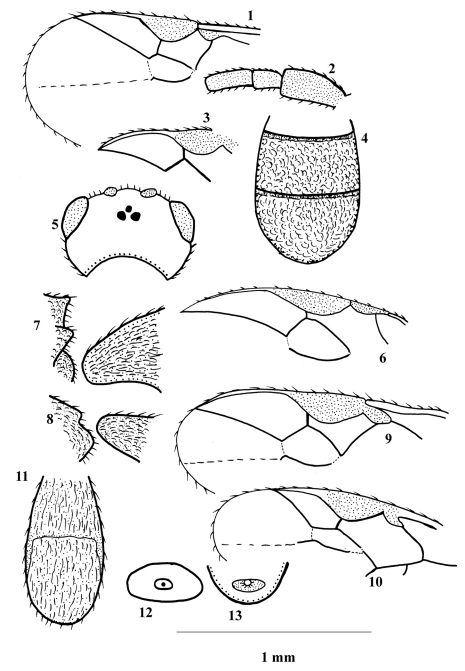
**1, 2** *Phanerotoma masiana* **3–5** *Phanerotoma dentata* **6** *Phanerotoma henedecasisella* **7** *Ascogaster quadridentata* **8** *Ascogaster excisa* **9** *Phanerotoma leucobasis* **10–12** *Chelonus basalis* (after Lozan and Tobias 2002) **13** *Chelonus sulcatus*. 1,3,6,9,10, part of fore wing (1.6 × scale line, 3.4 ×, 5.3 ×, 2.9 ×,1.9 × ); 2, basal flagellomeres (1.0 ×); 4,11, dorsal aspect of carapace (2.7 ×, 1.8 ×); 5, dorsalaspect of head (2.8 ×); 7,8, lateral aspect of propodeum (2.6 ×, id.); 12,13, apical aperture (1.8 ×, 2.2 ×).

**Figures 14–23.**
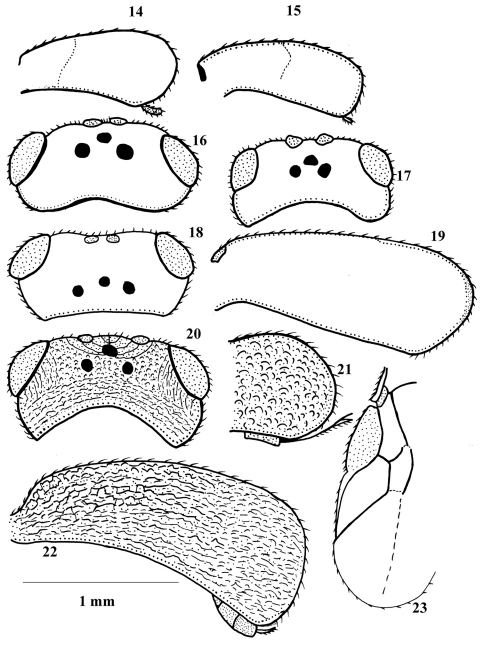
**14, 16** *Chelonus blackburni* **15, 17** *Chelonus curvimaculatus* **18, 19** *Chelonus obscuratus* **20, 22** *Chelonus inanitus* **21** *Chelonus oculator* **23** *Ascogaster quadridentata*. 14, 15, 19, 22, lateral aspect of carapace (4.8 × scale line, 5.0 ×, 2.0 ×, 2.1 ×); 16–18, 20, dorsal aspect of head (1.6 ×, id., id., 1.74 ×); 21, apical part of carapace (lateral aspect) (2.0 ×); 23, part of fore wing (2.1 ×).

**Figures 24–37.**
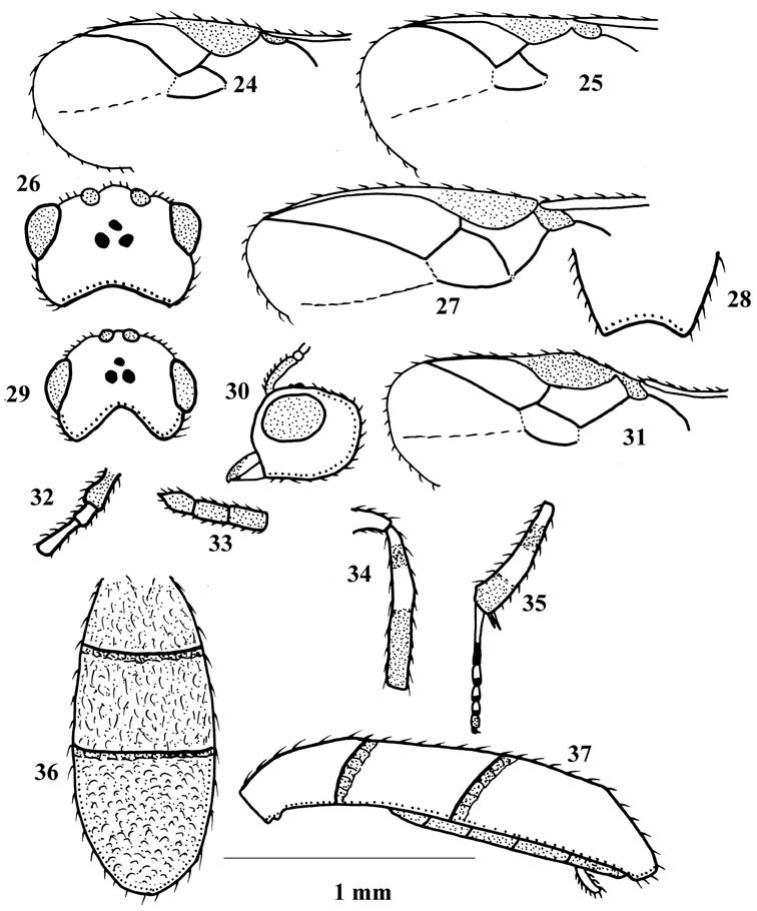
**24** *Chelonus inanitus* **25** *Chelonus oculator* **26–28** *Phanerotoma rufescens* **29–37** *Phanerotoma ponti*. 24, 25, 27, 31, part of fore wing (3.5 × scale line, 3.1 ×, 3.4 ×, 2.2 ×); 26, 29, dorsal view of head(3.2 ×, 3.3 ×); 28, apical part of carapace (dorsal aspect) (2.5 ×); 30, lateral aspect of head (6.0×); 32, basal flagellomeres (5.1 ×); 33, apical flagellomeres (3.1 ×); 34, middle tibia (1.7 ×); 35,part of hind leg (1.7 ×); 36, dorsal aspect of carapace (1.8 ×); 37, lateral aspect of carapace (2.5 ×).

**Figures 38–45.**
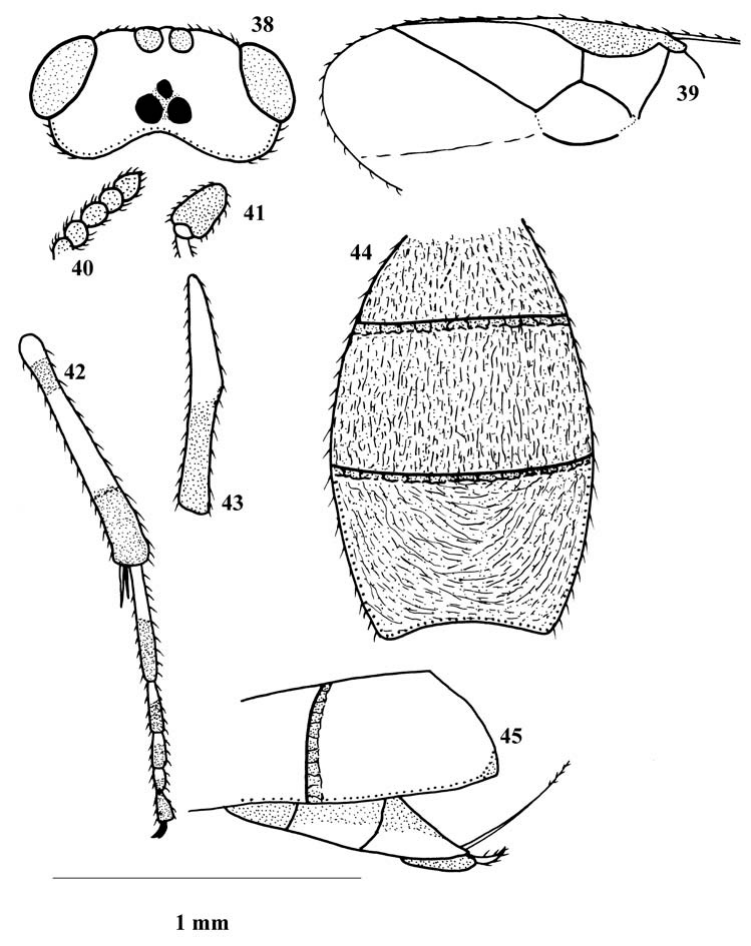
*Phanerotoma elbaiensis* **38** dorsal aspect of head (3.1 × scale line) **39** part of fore wing (3.1 ×) **40** apical flagellomeres (14.1 ×) **41** basal flagellomeres (14.1 ×) **42** part of hind leg (5.8 ×) **43** middle tibia (5.8 ×) **44** dorsal aspect of carapace (2.1 ×) **45** lateral aspect of carapace (2.0 ×).

##### * Phanerotoma (Phanerotoma) rufescens

(Latreille, 1809)

http://species-id.net/wiki/Phanerotoma_(Phanerotoma)_rufescens

Sigalphus rufescens Latreille 1809: 13.

###### Material.

1♀, Balteem (31°18'42.6954"N; 31°9'23.9394"E ), 13.VII.2010 [**CUC**].

###### Distribution.

New to Egypt,Europe (Central, Southwest and West) and Russia (Central, East and Northwest.

## Supplementary Material

XML Treatment for Ascogaster excisa

XML Treatment for Ascogaster quadridentata

XML Treatment for Chelonus (Chelonus) inanitus

XML Treatment for Chelonus (Chelonus) obscuratus

XML Treatment for Chelonus (Chelonus) oculator

XML Treatment for Chelonus (Microchelonus) basalis

XML Treatment for Chelonus (Microchelonus) blackburni

XML Treatment for Chelonus (Microchelonus) curvimaculatus

XML Treatment for Chelonus (Microchelonus) sulcatus

XML Treatment for Phanerotoma (Bracotritoma) masiana

XML Treatment for Phanerotoma (Bracotritoma) ponti

XML Treatment for Phanerotoma (Phanerotoma) dentata

XML Treatment for Phanerotoma (Phanerotoma) elbaiensis

XML Treatment for Phanerotoma (Phanerotoma) hendecasisella

XML Treatment for Phanerotoma (Phanerotoma) leucobasis

XML Treatment for Phanerotoma (Phanerotoma) rufescens
